# Clearance of beta-amyloid and tau aggregates is size dependent and altered by an inflammatory challenge

**DOI:** 10.1093/braincomms/fcae454

**Published:** 2024-12-14

**Authors:** Emre Fertan, Christy Hung, John S H Danial, Jeff Y L Lam, Pranav Preman, Giulia Albertini, Elizabeth A English, Dorothea Böken, Frederick J Livesey, Bart De Strooper, Rickie Patani, David Klenerman

**Affiliations:** Yusuf Hamied Department of Chemistry, University of Cambridge, Cambridge CB2 1EW, UK; UK Dementia Research Institute, University of Cambridge, Cambridge CB2 0XY, UK; The Francis Crick Institute, University College London, London NW1 1AT, UK; Department of Neuroscience, City University of Hong Kong, Kowloon 999007, Hong Kong SAR; Yusuf Hamied Department of Chemistry, University of Cambridge, Cambridge CB2 1EW, UK; UK Dementia Research Institute, University of Cambridge, Cambridge CB2 0XY, UK; Yusuf Hamied Department of Chemistry, University of Cambridge, Cambridge CB2 1EW, UK; UK Dementia Research Institute, University of Cambridge, Cambridge CB2 0XY, UK; VIB-KU Leuven Center for Brain & Disease Research, Herestraat 49, 0N5 box 602, 3000 Leuven, Belgium; VIB-KU Leuven Center for Brain & Disease Research, Herestraat 49, 0N5 box 602, 3000 Leuven, Belgium; Yusuf Hamied Department of Chemistry, University of Cambridge, Cambridge CB2 1EW, UK; UK Dementia Research Institute, University of Cambridge, Cambridge CB2 0XY, UK; Yusuf Hamied Department of Chemistry, University of Cambridge, Cambridge CB2 1EW, UK; UK Dementia Research Institute, University of Cambridge, Cambridge CB2 0XY, UK; Zayed Centre for Research into Rare Disease in Children, University College London, Great Ormond Street Institute of Child Health, London WC1N 1DZ, UK; VIB-KU Leuven Center for Brain & Disease Research, Herestraat 49, 0N5 box 602, 3000 Leuven, Belgium; UK Dementia Research Institute, University College London, London WC1E 6BT, UK; The Francis Crick Institute, University College London, London NW1 1AT, UK; Yusuf Hamied Department of Chemistry, University of Cambridge, Cambridge CB2 1EW, UK; UK Dementia Research Institute, University of Cambridge, Cambridge CB2 0XY, UK

**Keywords:** Alzheimer’s disease, conditioned media, induced pluripotent stem cell, single-molecule detection, super-resolution microscopy

## Abstract

Extracellular beta-amyloid aggregation and inflammation are in a complex and not fully understood interplay during hyperphosphorylated tau aggregation and pathogenesis of Alzheimer’s disease. Our group has previously shown that an immune challenge with tumour necrosis factor alpha can alter extracellular beta-sheet containing aggregates in human-induced pluripotent stem cell-derived cortical neurons carrying familial Alzheimer’s disease-related presenilin 1 mutations. Here, using single-molecule detection and super-resolution imaging techniques, we quantified and characterized the intra- and extracellular beta-amyloid and AT8-positive tau aggregates. Our results indicate a pre-existing Alzheimer’s disease-like pathology caused by the presenilin 1 mutation, with increased beta-amyloid aggregates in both the cell lysate and conditioned media compared to isogenic controls and also increased intracellular tau aggregates. The main effect of tumour necrosis factor alpha treatment on presenilin 1 neurons was the formation of larger intracellular beta-amyloid aggregates. In contrast, isogenic controls showed more significant changes with tumour necrosis factor alpha treatment with an increase in beta-amyloid aggregates in the media but not intracellularly and an increase in tau aggregates in both the media and cell lysate, suggesting a chronic inflammation-driven mechanism for the development of sporadic Alzheimer’s disease. Remarkably, we also found significant morphological differences between intra- and extracellular beta-amyloid and tau aggregates in human-induced pluripotent stem cell-derived cortical neurons, suggesting these neurons can only clear aggregates when small, and that larger aggregates stay inside the neurons. While majority of the beta-amyloid aggregates were cleared into the media, a greater portion of the tau aggregates remained intracellular. This size-dependent aggregate clearance was also shown to be conserved *in vivo*, using soaked and homogenized mouse and human post-mortem Alzheimer’s disease brain samples. As such, our results are proposing a previously unknown, size-dependent aggregate clearance mechanism, which can possibly explain the intracellular aggregation of tau and extracellular aggregation of beta-amyloid.

## Teaser

Fertan *et al*. show that inflammation can initiate an Alzheimer’s disease-like pathology in a stem cell model without prior disease phenotype and worsens the phenotype in disease models. Clearance of soluble aggregates is size dependent in cellular and mouse models, and human brain samples, with larger aggregates accumulating inside the cells.

## Graphical Abstract

**Graphical Abstract:**
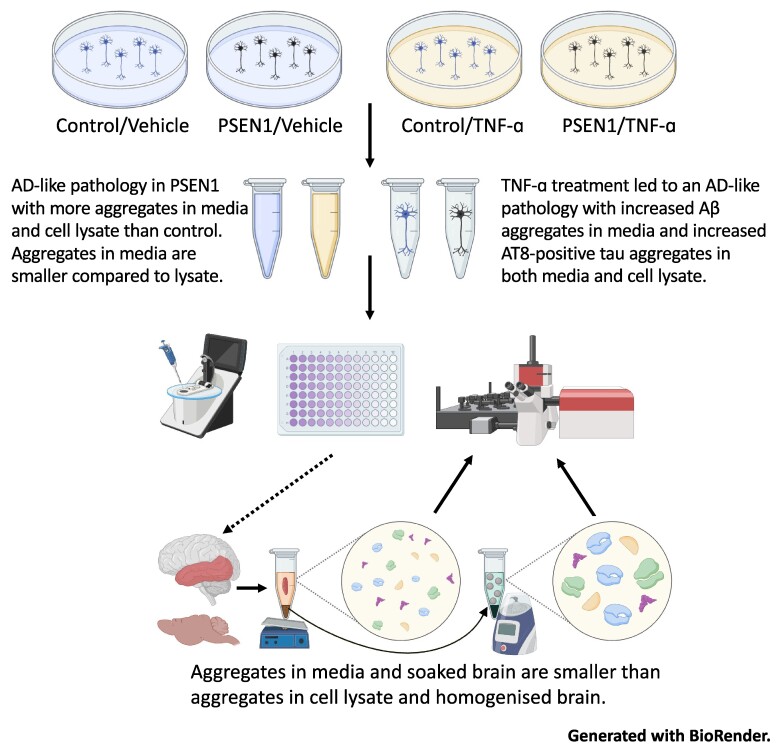

## Introduction

Alzheimer’s disease is the leading cause of dementia, with the neuropathological hallmarks of extracellular beta-amyloid (Aβ) and intracellular tau pathology, accompanied by gliosis and cerebral atrophy.^1,2^ While the Aβ and tau pathology are sometimes studied independently, growing evidence suggests that the small diffusible aggregates, often called oligomers, formed by these proteins promote the pathogenesis in Alzheimer’s disease in a synergistic manner.^3-5^ However, the small size of these aggregates, which is under the diffraction-limit of light, their low abundance, and high heterogeneity make them difficult to study using traditional methods such as immunohistochemistry or ELISA and require single-molecule detection.^6^ Indeed, we have recently showed Alzheimer’s disease-like pathology in a cerebral organoid model of chromosome 21 trisomy using single-molecule fluorescence microscopy, which was undetectable by traditional immunostaining methods.^7,8^

Amyloid precursor protein has a rapid turnover rate of about 30 min, after which its either processed in the membrane by alpha-secretase, producing non-amyloidogenic species, or by the endo-lysosomal system with beta- and gamma-secretase, producing Aβ.^9-11^ It is believed that most of the Aβ is extracellular, triggering a microglia-driven immune response, involving the release of cytokines including interleukins (IL) 1β and 6, tumour necrosis factor alpha (TNF-ɑ), and reactive oxygen species, through tyrosine kinase-based signalling.^12-14^ In return, these cytokines can accelerate Alzheimer’s disease pathology through both Aβ- and tau-related mechanisms.^15-17^ Indeed, TNF-ɑ inhibition has been shown to reduce tau hyperphosphorylation, neuronal loss and microgliosis in the PS19 mouse model of tauopathy.^16^ Meanwhile, intracellular aggregation of Aβ has also been observed^18-20^ and linked to altered signalling, oxidative stress and changes in gene expression.^21-23^ Recently, Hong *et al*.^24,25^ showed the existence of different soluble Aβ aggregate populations in the post-mortem Alzheimer’s disease brain, with the majority being less diffusible and require harsh extraction techniques and the remaining minority highly diffusible and can be extracted with gentle soaking. However, the morphological characteristics of these aggregates and their location in the brain remain elusive. On the other hand, most of the pathological tau aggregation is believed to be intracellular, following its detachment from the microtubules and hyperphosphorylation, leading to aggregation.^26,27^ However, to date, the morphological similarities and differences between the intra- and extracellular aggregates and the role of aggregate size on clearance are not understood.

Previously, our group has shown that 18 days of TNF-ɑ treatment can significantly increase extracellular, beta-sheet structure containing aggregates in human-induced pluripotent stem cell (hiPSC)-derived cortical neurons carrying familial Alzheimer’s disease-related presenilin 1-int4del (PSEN1^IN4^) mutation,^28,29^ which has been linked to endo-lysosomal dysfunction.^30^ However, aggregate imaging in this study was done using thioflavin T (ThT) and an aptamer, both binding to beta-sheet containing Aβ and alpha synuclein (aSyn) aggregates without specificity^31-33^; thus, it has not been possible to exclusively study Aβ. In the current study, we used the same hiPSC-derived cortical neuron model with their isogenic controls (ICs) and characterized the Aβ and AT8-positive (p)Tau aggregates using single-molecule pulldown (SiMPull) and DNA point accumulation for imaging in nanoscale topography (DNA-PAINT) with Aβ and AT8-positive tau-specific monoclonal antibodies, allowing us to directly compare the number, size and shape of the aggregates that are secreted into the media and accumulate in the neuron.

SiMPull was developed by Jain *et al*.^34^ to study protein complexes and uses a polyethylene glycol-pacified surface, coated with biotin. Following the application of neutravidin, biotinylated capture antibodies are used to specifically capture targets of interest. By using the same monoclonal antibody to detect the targets under total internal reflection fluorescence (TIRF), detection of aggregates (instead of monomers) is achieved. Using this method, our group previously studied human post-mortem brain samples from Alzheimer’s disease and Parkinson’s disease patients, as well as conditioned media samples from cerebral organoid models,^7,35,36^ characterizing the Aβ, tau and aSyn aggregates in these samples. By combining SiMPull with DNA-PAINT microscopy (Supplemental Fig. 1), super-resolution imaging of Aβ and phosphorylated tau aggregates can be performed. In DNA-PAINT, a short single DNA strand is conjugated to the detection antibody and the complimentary strand is conjugated to the fluorophore, causing rapid binding–unbinding events. This enables the super-resolution re-construction of targeted aggregates.^6,37,38^

Using these methods, we have been able to study the effects of a chronic inflammatory challenge on human cortical neurons with familial Alzheimer’s disease-related mutations and ICs, on both intracellular Aβ and tau aggregation, and the clearance of these aggregates into the media, providing novel insights into how neurons maintain protein homeostasis and how this is disrupted by inflammation. Meanwhile, by comparing the size of the intracellular and extracellular aggregates, we identified previously unknown relationships between aggregate size and neuronal clearance. Lastly, by morphologically characterizing soaked and homogenized samples from the APP^NL-G-F^ mouse model of Alzheimer’s disease, and post-mortem human brain samples, we tested the size dependence of cellular aggregate clearance *in vivo*.

## Materials and methods

### hiPSC differentiation to cortical neurons and TNF-ɑ treatment

We performed directed differentiation of human iPSCs into cortical neurons by seeding dissociated iPSCs on GelTrex-coated (Life Technologies, Cat. A1413301) six-well plates. The differentiation of hiPSCs to human cortical excitatory neurons was achieved by using a previously established method,^39^ and we have previously confirmed the cortical identity of iPSC-derived neuronal cultures using gene expression profiling.^33^ In brief, neural induction was initiated by replacing into a culture medium that supports neuronal differentiation and neurogenesis, a 1:1 mixture of N2- and B27-containing media (N2B27), supplemented with 1 μM dorsomorphin and 10 μM SB431542 to inhibit transforming growth factor beta (TGF-β) signalling during neural induction. The culture medium was refreshed every 24 h. At Day 12, neuroepithelial cells were harvested with dispase and replated in laminin-coated plates with fibroblast growth factor (FGF) 2-containing media for 4 days. Subsequently, FGF2 was withdrawn, and the cells were dissociated using Accutase (Thermo Fisher Scientific, Cat. 00-4555-56). The resulting neural progenitor cells were plated on GelTrex-coated plates. The plated neurons were maintained with 500 μL of fresh media every 2–3 days.

hiPSC-derived neurons carrying homozygous PSEN1^IN4^ mutation and ICs were generated by using CRISPR/Cas9 gene editing technique to knock in the mutation into the KOLF2.1 cell line (experiments are performed from one clone of each cell line in technical replicates). Neurons were treated daily with a cell media consisted of 1:1 mixture of N2 (DMEM/F12 + GlutaMAX, N2 supplement, 10 mg/mL human insulin, 100 mM sodium pyruvate, non-essential amino acids, 2-mercaptoethanol; Thermo Fisher Scientific, Cat. 10565018) and B27 (Neurobasal, B27 supplement, GlutaMAX, penicillin/streptomycin; Thermo Fisher Scientific, Cat. 21103049) media. Remaining (conditioned) media in the culture dish after 24 h was replaced with fresh media. Ten nanograms per millilitre of TNF-ɑ (PeproTech, Cat. 300-01A) treatment started on 60 days *in vitro* (DIV) and lasted for 18 days.

### Aβ peptide level analysis

For the analysis of extracellular Aβ peptide levels, Aβ38, Aβ40 and Aβ42 were measured in conditioned media on DIV 50, using multiplexed MesoScale Discovery assays on a QuickPlex SQ120 instrument [V-PLEX Plus Aβ Peptide Panel 1 (6E10) Kit, MesoScale Discovery, Cat. K15200G-1], following the manufacturer’s instructions.

### Cell pellet lysis

Following hiPSC-derived cortical neuron culturing, the cell pellets were stored in a −80°C freezer in 0.5 mL Eppendorf Protein LoBind^Ⓡ^ tubes (Eppendorf, Cat. 0030108094) until used in the experiments. Cell pellet lysing was performed by adding 100 µL lysis buffer consisted of PBS (Thermo Fisher Scientific, Cat. 10010023) with 1% Triton X-100 (Merck, cat. X100-100ML), protease (cOmplete^™^, Mini Protease Inhibitor Cocktail, Roche, Cat. 11836153001) and phosphatase (PhosSTOP^™^, Roche, Cat. 4906845001) inhibitors to the cell pellet and incubated on wet ice for 30 min, followed by centrifugation at 14 000 G for 10 min at 4°C. The cell lysis and conditioned media samples were kept in a −80°C freezer in 0.5 mL Eppendorf Protein LoBind^Ⓡ^ tubes until being used without additional freeze/thaw cycles to avoid sample degradation and modification.

### Sample normalization

Aggregate quantification from cell lysate and conditioned media samples can be confounded by the number of cells present in the sample producing and releasing those aggregates. Moreover, the efficiency of cell lysing also changes the harvested protein concentration. Thus, it is essential to normalize the data collected from these samples.

Following the homogenization of the hiPSC-derived cortical neuron pellet, a NanoDrop A_280_ analysis was performed, using a NanoDrop^™^ One/One^C^ Microvolume UV-Vis Spectrophotometer (Thermo Fisher Scientific, Cat. 701-058112), in order to determine the total protein concentration in the samples. While A_280_ is slightly less robust than other assays such as BCA in mixed samples, it was used due to the limited sample availability.

On the other hand, it is necessary to quantify the number of living cells releasing aggregates to the conditioned media samples, which is not possible with NanoDrop. In order to overcome this issue, glucose measures in the conditioned and unconditioned media samples were measured, and the glucose consumption was used as a factor of living cells, as described previously.^7^ In brief, this assay (abcam, Cat. ab65333) was performed by mixing 2 μL of media sample from each conditioned media sample, as well as unconditioned (fresh) media with 98 μL glucose assay buffer. Then, the samples were loaded to a 96-well plate in duplicates at 50 μL per well, alongside the assay standards, and 50 μL of reaction mix was added to each sample and standard well. After gently shaking the plate to allow mixing and incubating at 37°C for 30 min, protected from light, the plate was read at 570 nm absorbance.

### Tissue samples

Four (two male and two female), 6-month-old NL-G-F^ApoE3^ mouse brains and three (two male and one female) human post-mortem middle temporal gyrus (MTG) Braak stage 5 Alzheimer’s disease brains were used in this study. The NL-G-F mouse model of Alzheimer’s disease was developed to study Aβ pathology under endogenous expression levels with the familial Alzheimer’s disease-related Swedish, Iberian and Arctic mutations.^40^ Preman *et al.*^41^ crossed this model with apolipoprotein E (ApoE) knock-out mice and injected the offspring with an adeno-associated virus to mediate expression in astrocytes, to study the role of different isoforms. Mice expressing ApoE3 were used in this study as this isoform is considered neutral, in terms of Alzheimer’s disease pathogenesis. Mice were sacrificed at 6 months of age and perfused with PBS. The brains were removed from the skull, and then the cerebrums were detached from the cerebellum and snap frozen.

The post-mortem human brain samples from three Alzheimer’s disease cases—with no known history of neurological or neuropsychiatric symptoms other than Alzheimer’s disease, were acquired from the King’s College Brain Bank (with the approval of the London-Bloomsbury Research Ethics Committee, 16/LO/0508). The brain samples have been voluntarily donated without any compensation. The MTG segments were dissected and flash-frozen to be stored in −80°C freezers until processing. The average age of death of the donors was 84.3 ± 1.2 years, with an average post-mortem interval of 17.5 ± 6.7 h. ApoE genotype of the donors was 3/3, 3/4 and 3/4.

### Tissue sample preparation

The mouse and human brain tissue samples were received fresh-frozen (following PBS perfusion for the mice) and stored in −80°C freezers until processing by gentle soaking and homogenizing, as described by Hong *et al*.^24,25^ In brief, the artificial CSF soaking buffer was prepared by mixing 124 mM NaCl, 2.8 mM KCl, 1.25 mM NaH_2_PO_4_, 26 mM NaHCO_3_, 5 mM EDTA, 1 mM EGTA, 5 µg/mL leupeptin, 5 µg/mL aprotinin, 2 µg/mL pepstatin, 5 mM NaF, 20 µg/mL Pefabloc® SC protease inhibitor (Roche, Cat. 11585916001) and PhosSTOP^™^ phosphatase inhibitor tablet (Roche, Cat. 4906845001) and adjusting the pH to ∼7.3. One millilitre of buffer was added to 400 mg tissue (average mass of mouse cerebrum) in protein LoBind® tubes (Eppendorf, Cat. 0030108116) and placed on a HulaMixer^™^ (Thermo Fisher Scientific, Cat. 15920D) for 4 h at 4°C. The sample was first centrifuged at 2000 g for 10 min at 4°C, and the supernatant was separated from the pellet. Then the supernatant was centrifuged again, at 21 000 g for 2 h at 4°C, and the soaked samples were aliquoted and stored in −80°C. Meanwhile, the pellet from the first centrifugation was homogenized by first moving the tissue to 2 mL tubes prefilled with 1 mm zirconium beads (Scientific Labs, Cat. SLS1414) and adding 800 µL of homogenizing buffer (10 mM Tris-HCl, 0.8 M NaCl, 1 mM EGTA, 10% sucrose, 0.1% sarkosyl, Pefabloc® SC protease inhibitor and PhosSTOP^™^ phosphatase inhibitor tablet; pH ∼7.4) and processing the samples on an electronic tissue homogenizer (VelociRuptor V2 Microtube Homogeniser, Scientific Labs, Cat. SLS1401), at 5 m/s for two cycles of 15 s, with a 10-s gap in between, followed by centrifugation at 21 000 G for 20 min at 4°C. Then, the supernatant was collected and stored in a LoBind Eppendorf at 4°C, while the pellet was homogenized again by adding an additional 800 µL of homogenizing buffer and repeating the steps above. The supernatant from this step was mixed with the one from the previous step, aliquoted and stored in a −80°C freezer.

### SiMPull

Coverslips were prepared as described previously^35,36^ and kept in vacuumed containers at −20°C until used. The coverslip was first passivated with neutravidin (0.2 mg/mL) diluted in 0.05% PBS-T (v/v) (Tween20, Cat. P1379-25ML diluted in PBS) for 10 min. The neutravidin solution in each well was then removed, followed by washing with PBS-T (two cycles) and PBS with 1% Tween (one cycle)—hereon described as washing. For Aβ and AT8-positive tau, biotinylated 6E10 (BioLegend, Cat. 9340-02) and AT8 (Invitrogen, Cat. MN1020B) antibodies were used. Capture antibodies were diluted to 10 nM in PBS containing 0.1 mg/mL bovine serum albumin (BSA; Thermo Scientific, Cat. 10829410) and incubated for 15 min. Then, the wells were washed, and samples were incubated overnight at 4°C. In order to minimize non-specific binding, a blocking step was performed with solution containing 1 mg/mL BSA incubated for 30 min, before the capture antibody and after the sample incubation, followed by washing. For detection, the Alexa Fluor 647-labelled antibodies corresponding to the biotinylated capture antibody were diluted in BSA/PBS at 0.5, 2 and 1 nM concentrations and incubated for a duration of 45 and 15 min for 6E10 and AT8, respectively, followed by the washing step. Then, 7 µL of PBS was added to each well, and the coverslip was imaged using a purpose-built TIRF microscope^42^ using a 638 nm excitation laser. Fifty frames at an exposure time of 50 ms were recorded for at least 9 field of views (FoVs) per sample.

### DNA-PAINT

For super-resolution imaging, SiMPull coverslips were prepared as described above, but instead of the Alexa Fluor 647-labelled imaging antibody, a DNA-labelled antibody was introduced.^37,38^ After the washing steps, TetraSpeck microspheres (1:12 000 in TBS, 10 µL, Thermo Scientific, Cat. T7279) were introduced to each well for 10 min. The TetraSpeck solution was then removed, followed by a single wash with TBST, and a second PDMS gasket (Merck, GBL-103250-10EA) was stacked on the coverslip before introducing 4 µL of imaging strand (TGGTGGT- cy3B; atdbio) in TBS. Finally, the coverslip was sealed with another coverslip on top of the second PDMS gasket. Super-resolution imaging was conducted on the same microscope,^42^ with exposure time of 100 ms at 4000 frames, for at least 3 FoVs, using a 561 nm excitation laser.

### Statistical analysis

Two iPSC lines from independent donors were used in the cell culture experiments, alongside four (two male and 2 female) 6-month-old APP^NL-G-F^ mice and three (two male and one female) Braak stage 5, human post-mortem MTG (Brodmann area 21) tissues. Samples were encrypted with unique identification codes, and during the experiments, the investigators were blinded to sample details. Diffraction-limited imaging on SiMPull was performed as at least 9 FoVs on two independent wells (technical replicates). Super-resolution imaging was performed as at least 3 FoVs. Diffraction-limited images were reconstructed and analysed using ComDet v.0.5.5 plugin for ImageJ, and the super-resolution images were reconstructed, drift corrected and analysed using ACT^43^ and ASAP.^44^ The R Project Statistical Computing version 4.3.1 (2023-06-16)—‘Beagle Scouts’ was used for all statistical analyses, the graphs were generated in GraphPad Prism 7.0a for Mac OS X, and the cartoon figures were created with BioRender.com (agreement number: HN27IW29QQ for the Graphical Abstract and IA27IW2SP4 for Supplemental Fig. 1). Linear mixed effects models (LMEs) with the FoV and cell line included as a random variable were used to analyse the data [Akaike information criterion (AIC), likelihood ratio (LR) and probability (*P*) values were reported], and Kolmogorov–Smirnov tests were performed to compare the cumulative area distributions; 95% confidence intervals (CIs) were reported for *post hoc* analysis.

## Results

### TNF-ɑ exacerbates Alzheimer’s disease-like pathology in neurons with familial Alzheimer’s disease mutations and causes pathology in isogenic controls

Using cortical neurons derived from independent iPSC lines of two different donors with PSEN1^IN4^ mutations and their isogenic (CRISPR corrected) controls, we first studied the effect of chronic inflammatory stress induced by 18 days of 10 ng/mL TNF-ɑ treatment on Aβ and AT8-positive tau aggregate pathology. Overall, these analyses indicated that while chronic inflammation can promote Alzheimer’s disease-like pathology in neurons without a genetic predisposition, the pre-existing pathology is worsened by chronic inflammation in neurons with familial Alzheimer’s disease-related mutations.

### PSEN1^IN4^ mutation shifts Aβ production to longer species in hiPSC-derived cortical neurons

We first performed a multiplex ELISA, measuring total (monomer and aggregated) Aβ38, 40 and 42 levels, to confirm the same Aβ phenotype is formed in the PSEN1^IN4^ and IC hiPSC-derived cortical neurons as reported previously.^33^ The Aβ38 levels were decreased in the PSEN1^IN4^ neuron-conditioned media samples, there was no difference in Aβ40, and the Aβ42 levels were elevated, indicating the expected shift in Aβ production towards longer peptides (Supplemental Fig. 2). Importantly, the total Aβ42 concentration (monomer and aggregate) was below about 20 pM, indicating that the aggregate concentration is significantly lower than this value and below the detection limit of most traditional imaging methods, showing the utility of single-molecule techniques.

### Alzheimer’s disease-like pathology is present in PSEN1^IN4^ neurons prior to TNF-ɑ treatment

**Figure 1:**
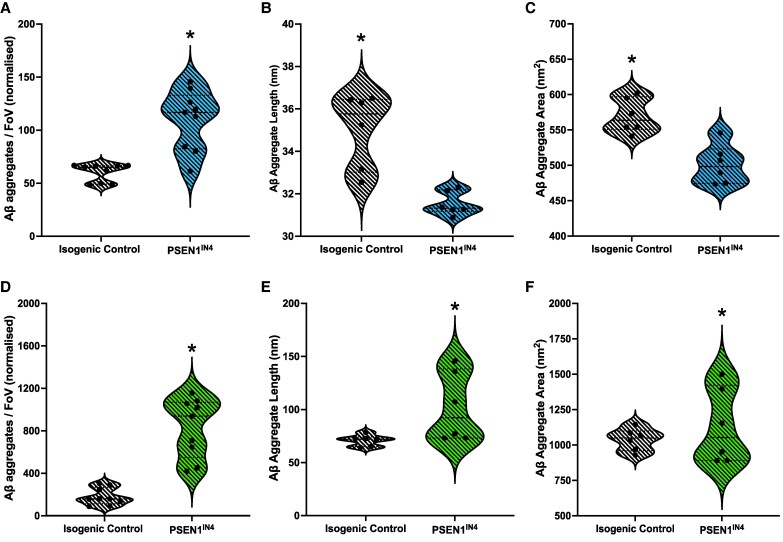
**Pre-treatment quantification and characterization of Aβ aggregates in the conditioned media and neuronal pellet lysates.** Diffraction-limited TIRF microscopy was used to count the Aβ aggregates in the conditioned media and neuronal pellet lysate. The size of these aggregates was also measured using super-resolution (DNA-PAINT) microscopy. Data for aggregate numbers were collected from iPSCs generated from two independent donors, and imaging was done for 9 FoVs per well, represented as individual data points on violin plots. Aggregate morphology was determined by analysing 17 664 aggregates from the PSEN1^IN4^ and 13 266 aggregates from the IC samples from the conditioned media samples (**B**, **C**) and 337 254 aggregates from the PSEN1^IN4^ and 151 815 aggregates from the IC samples from the neuronal lysates (**E**, **F**) over 3 FoVs. Differentiation and FoV were included in the LME analyses as random variables. (**A**) There were more Aβ aggregates in the PSEN1^IN4^-conditioned media samples than ICs (AIC = 671.3, LR = 4.79, *P* = 0.03, CI_95_ = 32.74, 66.71). (**B**) These aggregates were shorter in the PSEN1^IN4^-conditioned media samples than ICs (AIC = 60.0, LR = 14.42, *P* < 0,001, CI_95_ = 3.21, 4.23). (**C**) These aggregates were smaller in the PSEN1^IN4^-conditioned media samples than ICs (AIC = 131.7, LR = 13.55, *P* < 0.001, CI_95_ = 48.85, 89.66). (**D**) The PSEN1^IN4^ neurons contained higher number of Aβ aggregates than the ICs (AIC = 1163.1, LR = 16.30, *P* < 0.001, CI_95_ = 320.72, 977.97). (**E**) The PSEN1^IN4^ neurons contained longer Aβ aggregates than the ICs (AIC = 59.3, LR = 11.74, *P* = 0.001, CI_95_ = 37.62, 38.52). (**F**) The PSEN1^IN4^ neurons contained larger Aβ aggregates than the ICs (AIC = 79.9, LR = 7.23, *P* = 0.007, CI_95_ = 142.18, 159.02). * are used to indicate significant differences, determined by a *P* value under 0.05 and/or CI not containing 0.

On DIV 60 (prior to treatment), the amount of Aβ aggregates in the conditioned media samples was measured using SiMPull, using the amount of glucose in the media to normalize to the number of live neurons—a method that we have recently developed.^26^ The aggregate sizes and shapes (eccentricity) were then measured using DNA-PAINT. Aggregates of a range of sizes from 30 nm (resolution limited) to 400 nm were detected, although the majority of the aggregates were small, <40 nm in length. We found that the Aβ aggregate levels were significantly higher in the PSEN1^IN4^ media samples than the ICs (Fig. 1A), but the aggregates released into the media by the PSEN1^IN4^ neurons were shorter (Fig. 1B), smaller (Fig. 1C) and rounder than the IC neurons. Then, we measured the amount of Aβ aggregates in the cell pellets. PSEN1^IN4^ neuronal pellets contained significantly more aggregates (Fig. 1D), by a factor of ∼4, and these aggregates were also longer (Fig. 1E), larger (Fig. 1F) and more fibril-like than the intracellular Aβ aggregates in the IC neurons. Collectively, these results suggest that PSEN1^IN4^ mutation increases Aβ aggregation in hiPSC-derived cortical neurons leading to larger aggregates.

### Increased Aβ leads to tau pathology in PSEN1^IN4^ neurons

**Figure 2:**
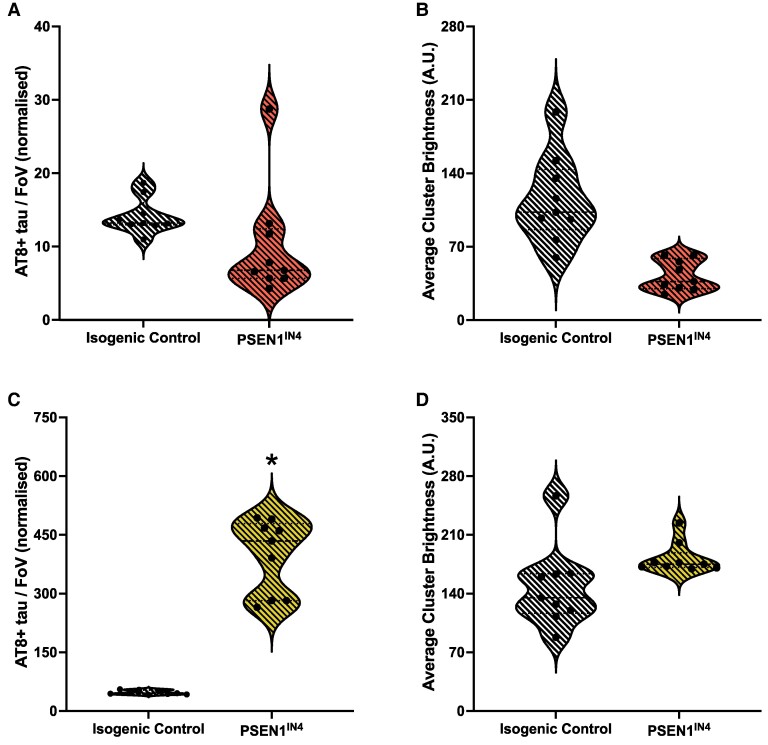
**Pre-treatment quantification and characterization of AT8-positive tau aggregates in the conditioned media and neuronal pellet lysates.** Diffraction-limited TIRF microscopy was used to count the AT8-positive tau aggregates in the conditioned media and neuronal pellet lysate. The size of these aggregates was compared using their brightness as a proxy for their size. Data for aggregate numbers were collected from iPSCs generated from two independent donors, and imaging was done for 9 FoVs per well, represented as individual data points on violin plots. Differentiation and FoV were included in the LME analyses as random variables. (**A**) The extracellular AT8-positive tau aggregates did not differ in number between the PSEN1^IN4^ and IC neurons (AIC = 582.2, LR = 1.83, *P* = 0.176, CI_95_ = −10.318, 2.05). (**B**) There were more pTau aggregates inside the PSEN1^IN4^ neurons than IC neurons (AIC = 497.5, LR = 43.37, *P* < 0.001, 251.92, 445.69). (**C**, **D**) The tau aggregate brightness, a measure of size, did not differ between PSEN1^IN4^ neurons than IC neurons in the conditioned media (AIC = 165.3, LR = 1.96, *P* = 0.16, CI_95_ = −44.08, 137.00) or intracellularly (AIC = 45.1, LR = 3.92, *P* = 0.05, CI_95_ = −200.16, 125.07). Brightness values are in arbitrary units (A.U.). * are used to indicate significant differences, determined by a *P* value under 0.05 and/or CI not containing 0.

The levels of AT8-positive tau aggregates were also measured in the conditioned media and cortical neuron pellet samples, prior to TNF-ɑ treatment. Larger aggregates will bind more antibodies and hence appear brighter when imaged. We therefore used the aggregate brightness as a proxy for aggregate size,^45^ due to limited sample availability. Neither the pTau levels (Fig. 2A) nor the aggregate brightness (Fig. 2B) differed in the media samples of the PSEN1^IN4^ hiPSC-derived cortical neurons compared to the IC neurons. On the other hand, the AT8-positive aggregate levels were significantly higher in the PSEN1^IN4^ neuron pellets (Fig. 2C), by a factor of 8, but with comparable brightness (Fig. 2D). Collectively, these results suggest that intracellular AT8-positive tau aggregation is a balance between pTau aggregate production and removal intracellularly and by actively or passively entering the media. The familial Alzheimer’s disease mutation shows increased intracellular pTau accumulation while the neurons without familial Alzheimer’s disease mutations can prevent the accumulation of tau aggregates more effectively by intracellular degradation and/or aggregates entering the media.

### TNF-ɑ-treated hiPSC-derived cortical neurons display decreased glucose consumption

**Figure 3:**
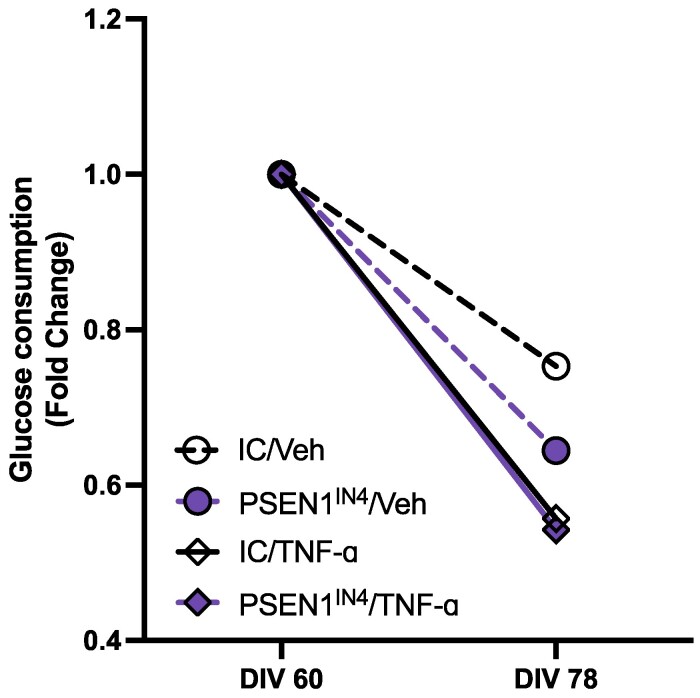
**Glucose normalization.** The amount of glucose remaining in the conditioned media samples was used to estimate the number living cells in the culture. A greater decrease in glucose consumption was observed in the TNF-ɑ-treated samples, indicating higher cell death. Data for glucose consumption were collected from iPSCs generated from two independent donors and the assay was run with two technical replicates, averaged and presented as data points on the graph.

We used the level of glucose in the media to monitor the number of live neurons following 18 days of TNF-ɑ treatment. The number of hiPSC-derived cortical neurons decreased over time in all cases. The reduction was 25% for the vehicle-treated control neurons, 36% for the vehicle-treated PSEN1^IN4^ neurons, 45% for the TNF-ɑ-treated control neurons and 47% for the TNF-ɑ-treated PSEN1^IN4^ neurons. The reduction was therefore significantly larger in the TNF-ɑ-treated samples, and the greatest change was observed in the TNF-ɑ-treated PSEN1^IN4^ hiPSC-derived cortical neurons (Fig. 3).

### TNF-ɑ treatment increases Aβ aggregate size in the PSEN1^IN4^ neurons

**Figure 4:**
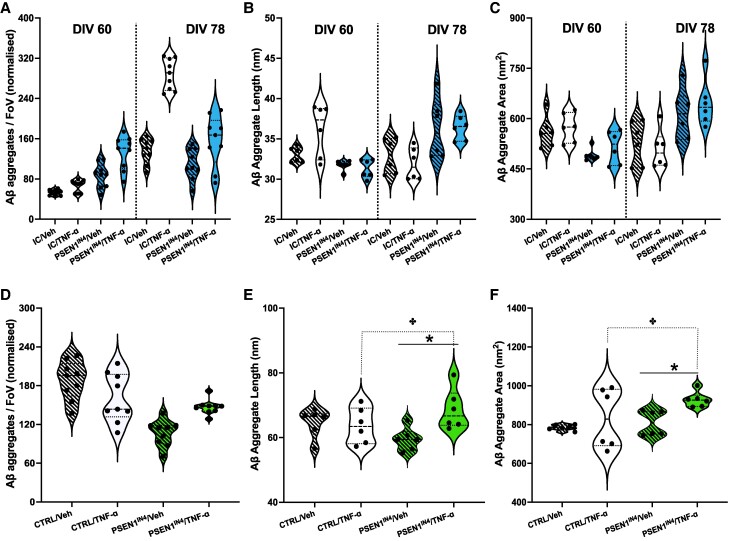
**Post-treatment quantification and characterization of Aβ aggregates in the conditioned media and neuronal pellet lysates.** Diffraction-limited TIRF and DNA-PAINT microscopy was used to count and characterize the aggregates following 18 days of TNF-ɑ or vehicle treatment of PSEN1^IN4^ and IC neurons. Data for aggregate numbers were collected from iPSCs generated from two independent donors, and imaging was done for 9 FoVs per well for two wells, represented as individual data points on the violin plots. Aggregate morphology was determined by analysing 33 184 aggregates from the PSEN1^IN4^ and 23 836 aggregates from the IC samples from the conditioned media samples (**B**, **C**) and 449 478 aggregates from the PSEN1^IN4^ and 228 055 aggregates from the IC samples from the neuronal lysates (**E**, **F**) over 3 FoVs and presented as violin plots, each data point on the violin plot representing an independent FoV. Differentiation and FoV were included in the LME analyses as random variables. (**A**) TNF-ɑ treatment increased the number of extracellular Aβ aggregate numbers in the IC neurons (AIC = 842.9, LR = 19.6, *P* < 0.001, CI_95_ = 133.41, 310.82). The aggregates increased in the PSEN1^IN4^ neurons, with a greater increase in for the TNF-ɑ-treated cells (AIC_Length_ = 132.0, LR = 25.57, *P* < 0.001, CI_95_ = 3.35, 6.83; AIC_Area_ = 289.8, LR = 21.40, *P* < 0.001, CI_95_ = 53.69, 208.50). (**B**) The length of the aggregates was smaller after TNF-ɑ treatment (AIC = 115.0, LR = 8.56, *P* = 0.003, CI_95_ = 0.80, 7.69). (**C**) The aggregate size was not altered (AIC = 266.8, LR = 0.81, *P* = 0.369). (**D**) The intracellular Aβ aggregate levels did not change between the groups but the PSEN1^IN4^ genotype (indicated by an asterisk) and TNF-ɑ treatment (indicated by a plus sign) independently increased the (**E**) aggregate length (AIC_Genotype_ = 149.54, LR = 4.68, *P* = 0.031, CI_95_ = 1.23, 36.52; AIC_Treatment_ = 76.1, LR = 4.35, *P* = 0.037, CI_95_ = 0.35, 9.53) and (**F**) aggregate area (AIC_Genotype_ = 218.3, LR = 3.41, *P* = 0.065, CI_95_ = −142.93, 4.76; AIC_Treatment_ = 139.0, LR = 25.20, *P* < 0.001, CI_95_ = 89.22, 142.34) with a greater TNF-ɑ-induced change in the PSEN1^IN4^ neurons. * and + signs are used to indicate significant differences, determined by a *P* value under 0.05 and/or CI not containing 0.

Following 18 days of TNF-ɑ treatment, Aβ aggregate levels were measured again in the conditioned media samples from the hiPSC-derived cortical neurons. Although the number of aggregates released from the PSEN1^IN4^ neurons did not change following 18 days of vehicle or TNF-ɑ treatment (Fig. 4A), both the length and area of these aggregates were greater following vehicle and, to a greater extent, TNF-ɑ treatment (Fig. 4B and C). On the other hand, Aβ aggregate numbers increased significantly in the IC-conditioned media samples following 18 days of treatment, with a greater increase in IC neurons treated with TNF-ɑ (Fig. 4A). While the size of the aggregates released by the IC cortical neurons receiving the vehicle remained the same (Fig. 4B and C), TNF-ɑ treatment decreased the length (Fig. 4B), but not the area (Fig. 4C) of the aggregates released by the IC neurons. These results show that in cortical neurons without familial Alzheimer’s disease-related mutations, there is an increase in the number of aggregates released into the media with age, without any increase in aggregate size, and this increase in aggregate number is enhanced by TNF-ɑ treatment. In contrast, for the PSEN1^IN4^ neurons, there is no change in the number of aggregates in the media, but an increase in the aggregate size with age, which is enhanced by TNF-ɑ treatment. For intracellular Aβ aggregates measured in the hiPSC-derived cortical neuron pellets following 18 days of treatment, there was no significant change in the number of aggregates (Fig. 4D). However, both the PSEN1^IN4^ and TNF-ɑ-treated neurons had bigger aggregates than IC and vehicle-treated neurons, respectively (Fig. 4E and F), suggesting an Alzheimer’s disease-like, genotype or treatment-dependent increase in Aβ aggregation. The changes in aggregate size on treatment by TNF-ɑ are more clear when we look at the cumulative length distribution. While there is no change in the IC neurons (Supplemental Fig. 3A), there is a clear increase in larger aggregates in the PSEN1^IN4^ neurons (Supplemental Fig. 3B).

Overall, these data show that IC neurons prevent increased intracellular aggregation when exposed to TNF-ɑ by secreting more Aβ aggregates into the media. In contrast, the PSEN1^IN4^ neurons are unable to increase the number of aggregates in the media and this leads to an increase in the size of the intracellular Aβ aggregates.

### TNF-ɑ induces the release of AT8-positive tau aggregates in the PSEN1^IN4^ neurons but not in ICs

**Figure 5:**
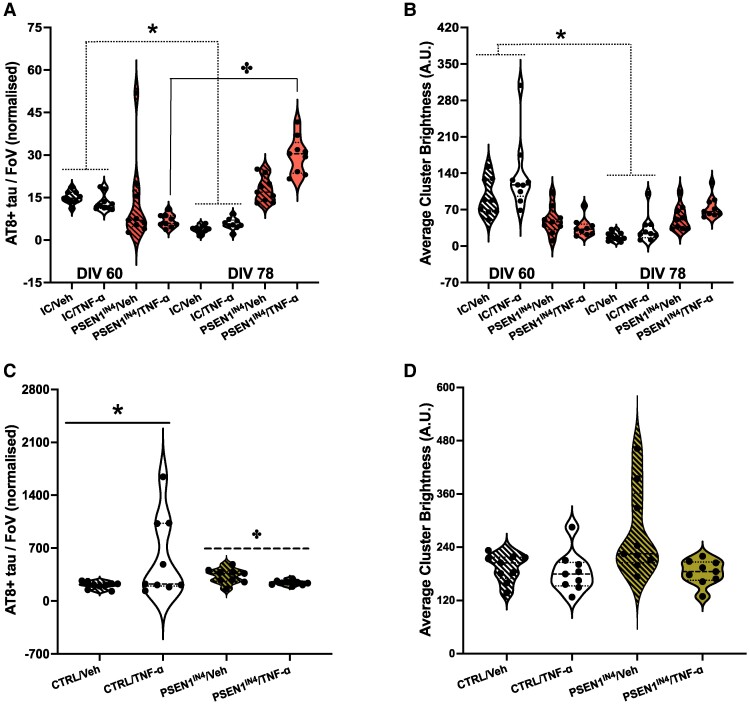
**Post-treatment quantification and characterization of AT8-positive tau aggregates in the conditioned media and neuronal pellet lysates.** Diffraction-limited TIRF microscopy was used to count and characterize the aggregates following 18 days of TNF-ɑ or vehicle treatment of PSEN1^IN4^ and IC neurons. Data for aggregate numbers were collected from iPSCs generated from two independent donors, and imaging was done for 9 FoVs per well for two wells, represented as individual data points on violin plots. Differentiation and FoV were included in the LME analyses as random variables. (**A**) The AT8-positive tau aggregate levels in the conditioned media for the IC neurons decreased with both treatments (AIC = 485.4, LR = 59.93, *P* < 0.001, CI_95_ = 6.58, 12.32). The number of aggregates increased in the TNF-ɑ-treated PSEN1^IN4^-conditioned media samples (AIC_Amount_ = 618.0, LR = 6.76, *P* = 0.001, CI_95_ = 11.91, 34.67). (**B**) The tau aggregate brightness decreased in the IC-conditioned media samples for both treatments (AIC = 168.6, LR = 5.85, *P* = 0.016, CI_95_ = 8.57, 12.05). (**C**) TNF-ɑ treatment decreased the intracellular pTau aggregates in the PSEN1^IN4^ neurons, and levels were increased in the ICs (AIC = 884.2, *F* = 4.74, *P* = 0.033). (**D**) Intracellular pTau aggregate brightness, as a proxy for size, did not change significantly with treatment in the IC neurons (AIC = 86.9, *F* = 0.10, *P* = 0.748), but the AT8-positive tau aggregates that were found inside the TNF-treated PSEN1^IN4^ neurons were less bright (AIC = 91.6, *F* = 3.90, *P* = 0.048). Brightness values are in arbitrary units (A.U.). * and plus + signs are used to indicate significant differences, determined by a *P* value under 0.05 and/or CI not containing 0.

Eighteen days of treatment decreased the AT8-positive tau aggregate levels in IC conditioned media samples regardless of treatment type (Fig. 5A), accompanied by a decrease in aggregate size, as measured by brightness. This was exacerbated by TNF-ɑ treatment (Fig. 5B). Meanwhile, AT8-positive tau aggregate levels (Fig. 5A) were increased in the TNF-ɑ-treated PSEN1^IN4^-conditioned media samples, without a change in brightness (Fig. 5B), and did not change in the vehicle-treated PSEN1^IN4^-conditioned media samples (Fig. 5A and B). On the other hand, TNF-ɑ-treated IC neurons had significantly higher intracellular pTau levels compared to the vehicle-treated IC neurons, while the levels were decreased in the TNF-ɑ-treated PSEN1^IN4^ neurons (Fig. 5C). Meanwhile, the brightest AT8-positive tau aggregates were found inside the PSEN1^IN4^ neurons treated with the vehicle. The brightness decreased significantly by TNF-ɑ treatment in this group and remained consistent in the IC neurons (Fig. 5D). This suggests that while 18 days of TNF-ɑ treatment promote pTau pathology in neurons with familial Alzheimer’s disease-related mutations and ICs, the PSEN1^IN4^ neurons cleared the AT8-positive tau aggregates more efficiently.

### Aβ and AT8-positive tau aggregate release is size dependent

Using single-molecule detection and super-resolution microscopy, we compared the size of the aggregates in the hiPSC-derived cortical neuron pellets and conditioned media samples and estimated the ratio of the aggregates cleared from the neurons.

### hiPSC-derived cortical neurons release smaller aggregates, but retain larger ones

**Figure 6:**
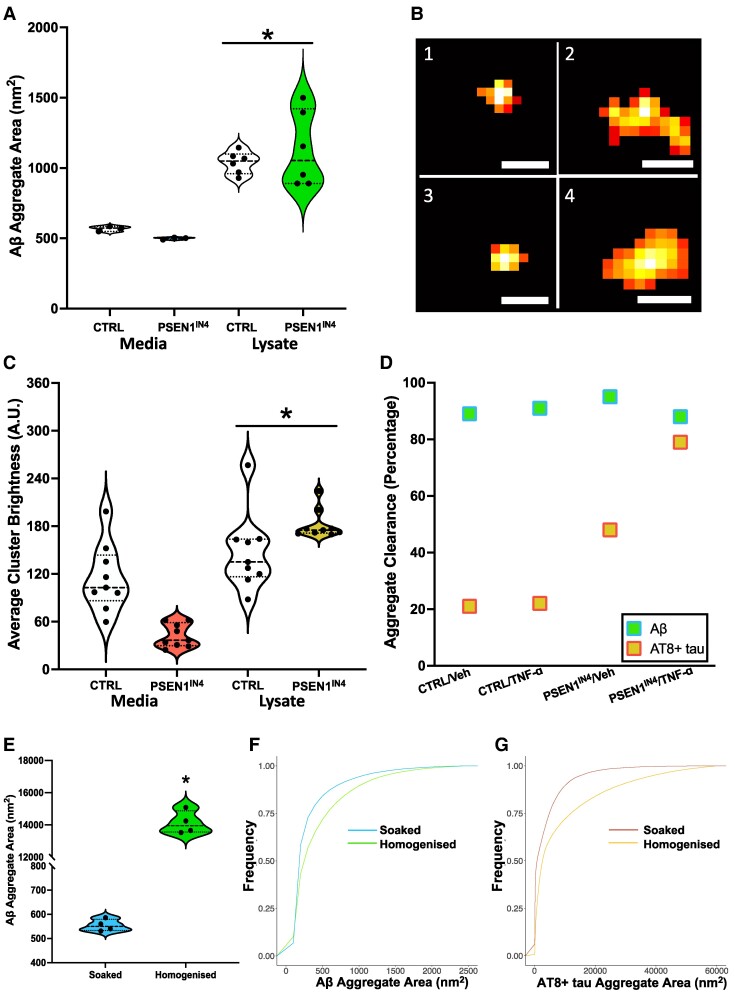
**Size comparison and clearance rates of aggregates.** (**A**) Comparison of the area of the Aβ aggregates in the conditioned media and hiPSC lysate samples using 6E10 antibody and DNA-PAINT microscopy using data from 489 069 Aβ aggregates from the neuronal lysates and 30 930 Aβ aggregates from the conditioned media were analysed and compared using 95% CIs (CI_95_Aβ = 444.74, 707.92); data points on violin graph represent individual FoVs. (**B**) Example super-resolution images. Aβ aggregate images from the vehicle-treated extra- (1) and intracellular (2) and TNF-ɑ-treated extra- (3) and intracellular (4) hiPSC-derived human cortical neuron samples (scale bars are 50 nm). (**C**) Comparison of the area of pTau aggregates in the conditioned media and neuronal lysate using 3781 AT8-positive tau aggregates from the neuronal lysates and 3724 AT8-positive tau aggregates from the conditioned media were compared using 95% CIs (CI_95_Tau = 48.01, 169.97) and presented as a violin plot, with each point on the plot representing a FoV; brightness values are in arbitrary units (A.U.). (**D**) Estimated aggregate clearance from the cultures calculated over the eight media changes for the two independent cell lines over 18 days. Aβ aggregates showed higher clearance levels than the pTau aggregates in all groups. (**E**) Average aggregate area plot of the Aβ aggregates from soaked and homogenized APP^NL−G−F^ mouse brain showing a size difference (CI_95_ = 12 880.77, 13 286.09). Data were collected from four APP^NL−G−F^ (two male and two female) mice; average per mouse represented as a data point in the violin graph. (**F**, **G**) Cumulative area plots of the Aβ and AT8-positive tau aggregates for soaked and homogenized human Alzheimer’s disease post-mortem brain samples. Kolmogorov–Smirnov tests were used to check if the area distributions are separated significantly, and then 95% CIs were performed to check if the average aggregate area differed. Both distributions for (**F**) Aβ (*D* = 0.06, *P* < 0.001; calculated using data from 71 603 aggregates) and (**G**) AT8-positive tau (*D* = 0.12, *P* < 0.001; calculated using data from 81 546 aggregates) differed significantly between the soaked and homogenized samples, and the average aggregate area difference also reached significance for AT8-positive tau (CI_95_Aβ = −123.10, 279.15; CI_95_Tau = 7902.79, 12 463.25). Data were collected from three (two male and one female), Braak stage 5, MTG samples. * are used to indicate significant differences, determined by a *P* value under 0.05 and/or CI not containing 0.

When we compared the extracellular Aβ aggregates in the conditioned media to the intracellular aggregates in the neuronal pellet, we found that the area of the intracellular Aβ aggregates was significantly larger than the ones in the conditioned media (CI_95_ = 444.74, 707.92; Fig. 6A), with the intracellular aggregates being larger for the PSEN1^IN4^ than the IC neurons (see Fig. 6B and Supplemental Fig. 4A–D for sample Aβ aggregate images). In particular when we look at the length and area distributions of the aggregates as shown in Supplemental Fig. 4E and F, we observe a significant fraction of Aβ aggregates longer and larger than 100 nm and 1000 nm^2^ in the lysate, while most of the aggregates are <40 nm and 600 nm^2^ in the media.

Similar to Aβ, AT8-positive tau clearance was also shown to be size dependent, as the intracellular aggregates from the neuronal pellets were brighter than the aggregates in the conditioned media samples (CI_95_ = 48.01, 169.97; Fig. 6C). These results suggest that hiPSC-derived cortical neurons can only release and hence remove smaller aggregates into the media, up to a length of on average 35 nm and area of on average 550 nm^2^. Consequently, aggregates larger than this size and area will accumulate intracellularly, and this occurs more in the PSEN1^IN4^ hiPSC-derived cortical neurons.

### Aβ is cleared more efficiently than pTau

Then, we estimated the ratio of intra- and extracellular aggregates by comparing the aggregate numbers and mass from the conditioned media and neuronal pellets (Fig. 6D). This was done by using the aggregate number and size measured in the samples to predict the total amount intracellularly (by using the total sample volume), and extracellularly (by using the total volume and number of DIV). This analysis showed that both the TNF-ɑ and vehicle PSEN1^IN4^ and IC neurons cleared >98% of the Aβ aggregates they produced, over the duration of the experiment, and only a small portion remained inside by the end. When analysed by aggregate mass instead of numbers, assuming the aggregates were all spherical—which is a lower limit, the highest clearance was shown in the vehicle-treated PSEN1^IN4^ neurons (95%), followed by TNF-ɑ-treated IC neurons (91%), vehicle-treated IC neurons (89%) and lastly the TNF-ɑ-treated PSEN1^IN4^ neurons (88%). These results show that most of the Aβ aggregates formed by the hiPSC-derived neurons are released to the extracellular space.

On the other hand, a greater portion of the AT8-positive tau aggregates were intracellular. By aggregate numbers, the greatest clearance was in the TNF-ɑ-treated PSEN1^IN4^ neurons (90%), followed by the vehicle-treated PSEN1^IN4^ neurons (88%), vehicle-treated IC (77%) and least in the TNF-ɑ-treated IC neurons (61%).When analysed by mass, again assuming the aggregates are all spherical, the differences were even more noticeable with again the greatest clearance in the TNF-ɑ-treated PSEN1^IN4^ neurons (79%), followed by the vehicle-treated PSEN1^IN4^ neurons (48%), TNF-ɑ-treated IC (22%) and least in the vehicle-treated IC neurons (21%). Alongside the size measures, these results collectively show that the majority of the Aβ aggregates are small and cleared from the hiPSC-derived neurons. Meanwhile, the pTau aggregates, which are larger, are cleared less efficiently from the neurons (Fig. 6D).

### Size-dependent aggregate clearance is preserved *in vivo*

It has been previously shown that gentle soaking and harsher homogenizing of the brain tissue harvest different subtypes of the soluble aggregates, with the easily diffusible soaked aggregates being more toxic.^24^ Moreover, size exclusion chromatography has shown a greater amount of larger molecular weight Aβ in the homogenized samples, while the soaked samples contained a greater amount of low molecular weight Aβ.^24^ However, to the best of our knowledge, the exact sizes of the Aβ and pTau aggregates in the soaked and homogenized fractions have not been determined. Here, we used the same tissue processing method on APP^NL-G-F^ mice with astrocytes expressing human ApoE3 and human post-mortem Alzheimer’s disease brain samples to compare the size of the aggregates in the soaked and homogenized samples. The NL-G-F mice with ApoE3 was used in these experiments, given the neutral role of this isoform on Alzheimer’s disease pathogenesis, unlike protective ApoE2 and detrimental ApoE4.^46^

The average area of the Aβ aggregates was significantly larger (CI_95_ = 12 880.77, 13 286.09) in the homogenized samples from the NL-G-F mouse brain compared to the soaked samples (Fig. 6E). Meanwhile, in the post-mortem human Alzheimer’s disease brain samples, the areas of the Aβ (*D* = 0.06, *P* < 0.001) and AT8-positive tau (*D* = 0.12, *P* < 0.001) aggregates were larger in the homogenized samples, indicated by a rightward shift in the cumulative area distribution plots (Fig. 6F and G), but the difference in average aggregate area only reached significance for the tau aggregates (CI_95_Aβ = −123.10, 279.15; CI_95_pTau = 7902.78, 12 463.25). It should be noted that the brain samples were serially extracted, first soaked and then same sample homogenized. As such, these results collectively show that the more readily diffusible Aβ and pTau aggregates in the brain, which can be extracted by gentle soaking, are smaller in size, while harsher homogenization with mechanical and chemical (detergent) disruption of the cellular integrity is needed to harvest the larger aggregates, suggesting that the cellular aggregate clearance in the brain may also be size dependent.

## Discussion

This study investigated the relationships between Alzheimer’s disease pathogenesis and neuroinflammation, as well as how the clearance of the small-diffusible aggregates depends on their size. Since aged microglia are more prone to activation and releasing inflammatory cytokines,^47^ their stimulation due to immune challenges can impact neurons and conceivably trigger Alzheimer’s disease, even in the absence of previous pathology.^48^ Similar trends have also been reported in other amyloid-related dementias, such as familial British dementia, during which systemic inflammation can lead to acute amnesia.^49^ On the other hand, Aβ can also trigger microglia and increase the release of pro-inflammatory cytokines,^13,14^ which in turn accelerate Alzheimer’s disease progression. In order to understand the changes in Aβ and phosphorylated tau aggregation with and without pre-existing Alzheimer’s disease-like pathology, we treated hiPSC-derived PSEN1^IN4^ mutant cortical neurons and their isogenic controls (ICs) with TNF-ɑ for 18 days.

In a previous study using a similar design, we have shown increased beta-sheet containing aggregate release following TNF-ɑ treatment from hiPSC-derived cortical neurons with PSEN1 mutations; meanwhile, the levels did not differ from their ICs prior to treatment.^33^ However, that study was limited to studying aggregates in the media alone, without investigating the intracellular aggregates. Moreover, the beta-sheet containing aggregates consisted of both Aβ and aSyn. Here, we quantified and characterized Aβ and AT8-positive tau aggregates extracellularly in the conditioned media and intracellularly in neuronal pellets.

By 60 DIV (prior to TNF-ɑ treatment), the PSEN1^IN4^ neurons had increased intra- and extracellular Aβ aggregates and increased intracellular AT8-positive tau aggregates compared to isogenic controls. However, it is worth noting that the controls also had both Aβ and tau aggregates, suggesting that aggregates form in control neurons but do not accumulate since their rate of formation is balanced by their rate of removal, while the PSEN1^IN4^ neurons showed more intracellular aggregates due to an imbalance between production and removal. Notably, the Aβ aggregates released by the PSEN1^IN4^ neurons were smaller than those released by IC counterparts, with larger aggregates remaining inside the neurons. Importantly, in the case of both the PSEN1^IN4^ and IC neurons, the Aβ aggregates found in the conditioned media were significantly smaller than the ones found in the cell lysate, suggesting that the source of these aggregates is clearance by the cells, rather than release following cell death, and hiPSC-derived cortical neurons are more effective at clearing smaller aggregates. The PSEN1^IN4^ cells also had higher amounts of intracellular AT8-positive tau, suggesting that the early Aβ aggregation promotes tau pathology in this model. While PSEN1 mutations have been previously linked to tau pathology,^50,51^ to the best of our knowledge, this is the first study showing AT8-positive tau aggregate pathology caused by the PSEN1^IN4^ mutation. This link between Aβ-related mutations and tau pathology agrees with the previous findings of *APP* mutations correlating with pathological tau aggregation in iPSC-derived neuronal models of Alzheimer’s disease,^52^ not only showing that iPSC-derived neuronal models are valuable models to study the pathophysiology of Alzheimer’s disease but also supporting the amyloid cascade hypothesis.

Following 18 days of TNF-ɑ treatment, the sizes of the Aβ aggregates produced and released by the PSEN1^IN4^ neurons were significantly increased, without a difference in aggregate numbers. While TNF-ɑ can increase total Aβ production by increasing *APP* expression,^53^ it can also alter β-^54^ and γ-secretase^55^ activity, thus change the Aβ species produced. It has been shown that Aβ peptides at different lengths and with different mutations differ in aggregation kinetics and dynamics,^56,57^ leading to the formation of oligomeric species at different sizes with different mechanisms of toxicity.^58,59^ In particular, larger aggregates are more inflammatory.^60^ AT8-positive tau levels were also altered in the PSEN1^IN4^ neurons following TNF-ɑ treatment with increased release into the media and decreased intracellular levels. This may be due to the combination of inflammation-mediated tau phosphorylation and Aβ-driven tau release from the neurons. Collectively, our results suggest that the pre-existing Aβ pathology in the PSEN1^IN4^ neurons is aggravated with the TNF-ɑ treatment, leading to the accumulation of larger Aβ aggregates intracellularly, but no clear changes in tau pathology.

There are notable differences compared to our previous work, Whiten *et al.*,^33^ where TNF-ɑ treatment led to an increase in the number of beta-sheet containing aggregates in the PSEN1^IN4^ neurons and these aggregates were slightly smaller than the non-treated neurons. This can be due to the previous work measuring both Aβ and aSyn aggregates, without differentiating them. Alternatively, we may be observing accelerated pathology in these current experiments, despite using isogenic neurons. In support of this concept, our group^7^ and others^61^ have shown differences in the rate of pathogenesis in isogenic hiPSC-derived Alzheimer’s disease models, where the same pathogenic pathway is followed but at a faster or slower rate for different cell preparations.

Remarkably, Alzheimer’s disease-like pathological changes were also observed in the IC neurons following TNF-ɑ treatment, as these cells released a greater number of smaller Aβ aggregates. Since the intracellular Aβ numbers were not increased, these results suggest that even though TNF-ɑ treatment can increase Aβ production in non-Alzheimer’s disease neurons, these aggregates can be cleared, as the clearance mechanisms of the cells are not yet exhausted. These results also show the importance of single-molecule and super-resolution imaging techniques, since the aggregates formed by the IC neurons following treatment were on average 31 nm in length, significantly under the resolution limit of most immunofluorescence methods.^6^ On the other hand, increased intracellular AT8-positive tau accumulation was evident in the IC neurons, following TNF-ɑ treatment. Therefore, in contrast to the PSEN1^IN4^ cells, the IC neurons have no pre-existing Aβ or tau pathology, but TNF-ɑ treatment increases the tau pathology (but not intracellular Aβ aggregates) and cell loss, as measured by glucose consumption. This suggests that chronic inflammation is an alternative mechanism to increased intracellular Aβ, to promote pTau aggregation. In the Alzheimer’s disease brain, the presence of increased extraneuronal Aβ aggregates causes an inflammatory response in astrocytes^62^ and microglia,^63^ in turn increasing intraneuronal tau aggregation and hence creating a positive cycle that drives disease pathogenesis.

Another central finding of this study is the significant size and concentration difference between intracellular and extracellular Aβ and pTau aggregates, suggesting that hiPSC-derived cortical neurons can only secrete aggregates up to a certain size (in this case on average 550 nm^2^). To the best of our knowledge, this is the first study reporting a significant size difference in the cell pellets (intracellular) and conditioned media samples (extracellular), and these findings can be helpful to further understand the aggregate clearing capabilities of neurons. Other work has also shown that Aβ accumulation may happen both intra-^19^ and extracellularly,^64^ involved in different pathological mechanisms.^65^ Our results suggest that >95% of the Aβ aggregates produced by the hiPSC-derived cortical neurons are small and thus released to the extracellular space, while only a small portion made of larger aggregates remain inside. While most of the Aβ processing and early aggregation occurs in the endo-lysosomal structures, which are then exocytosed, some leakage occurs due to the loss of membrane impermeability, leading to an aggregation in the cytosol and other organelles causing cellular stress^66,67^ and formation of larger intracellular aggregates. *In vivo*, the majority of the small aggregates released from the endo-lysosomes may be cleared from the brain by the glymphatic system,^68^ but the cellular system may get saturated over time, as we have shown here with the PSEN1^IN4^ mutation and chronic inflammation.^69^ On the other hand, more and larger aggregates accumulating inside the neurons may cause mitochondrial dysfunction and synaptic damage^70^ and altered gene expression.^23,71^ Concurrently, a greater portion of AT8-positive tau aggregates remain inside the neurons, possibly due to the formation of larger aggregates in the cytosol.^72^ It has previously been shown that most of the tau released from cultured human neurons and in the central nervous system is C-terminal truncated and often lacking the microtubule binding region (MTBR), which is required for aggregation, and thus monomeric.^73^ However, a small amount of aggregated tau has been detected in the conditioned cell media and CSF, which contain the MTBR, and thus formed inside the neurons, which has been suggested to be a biomarker for tau pathology in Alzheimer’s disease.^74^ By using aggregate-specific methods and the AT8 antibody, we specifically studied these small amounts of aggregated pTau species in the media, which are formed in the neurons, and compared them to the intracellular aggregates, which we found to have a significantly larger size distribution. These intracellular pTau aggregates may disrupt cellular signalling^75^ and DNA repair.^76^ As such, the mechanism for aggregate clearance may be active or passive and differ between Aβ and tau. If passive, then it may require too much energy to move large aggregates through the cell membrane into the media leading to a size cut-off.

We also studied the aggregates in the APP^NL-G-F^ mouse model of Alzheimer’s disease, with astrocytes expressing ApoE3, and post-mortem human MTG samples from donors with Alzheimer’s disease using gentle soaking, to remove the readily available aggregates followed by homogenization. The aggregates obtained by soaking are not directly equivalent to those present in the media of neuronal cultures since they are still intracellular, but easily diffusible. On the other hand, aggregates in the homogenate are more directly comparable to the cell lysate. Despite this potential difference, the aggregates produced by gentle soaking were significantly smaller than the ones further extracted by homogenization. Notably, the size difference was smaller in the human brain samples, with an increase in the size of the aggregates in the soaked portion. Since these samples were from Braak stage 5 brains, which show significant temporal lobe atrophy,^61,62^ the larger aggregates in the soaked samples may be related to the release of aggregates due to neuronal death. As such, these results also suggest that possibly the disruption of the neuronal integrity due to apoptosis or necrosis at later disease stage leads to the release of larger aggregates and the formation of plaques. Overall, our results from the hiPSC-derived neurons combined with the mouse and human brain samples support size-dependent clearance mechanisms for Aβ and pTau aggregates, which has been previously unknown.

## Conclusions

These findings suggest that PSEN1^IN4^ neurons have high levels of intracellular soluble pTau aggregates, prior to any inflammatory treatment, and the effect of the treatment is to increase the amount of intracellular soluble Aβ aggregates. In contrast, IC neurons have low levels of intracellular Aβ due to more effective active or passive secretion of Aβ aggregates into the media and inflammatory treatment leads to increased intracellular tau aggregates and extracellular Aβ aggregates, resembling Alzheimer’s disease pathology. Taken together, these findings suggest that intracellular tau aggregation, the key hallmark of Alzheimer’s disease, may be caused by various mechanisms. This also provides a possible explanation for how sporadic Alzheimer’s disease develops and it would be informative to expose IC neurons to TNF-α for longer times in future work to see how the pathology develops.

Importantly, we observed that both healthy and Alzheimer’s disease hiPSC-derived cortical neurons produce Aβ aggregates and can only secrete smaller aggregates into the media. We also estimated that over 95% of the Aβ aggregates are released from the hiPSC-derived neurons, while a greater portion of the pTau aggregates remain inside, possibly due to their larger size. While these findings are novel and suggest a size-dependent clearance mechanism for both Aβ and pTau aggregates, they also open up multiple avenues for future studies. The exact clearance mechanisms of Aβ and pTau, the different roles of intra- and extracellular aggregates in Alzheimer’s disease pathogenesis and the cellular localization of the larger aggregates remaining inside the neurons need to be determined and their link to neuroinflammation understood. Here, we used a TNF-ɑ challenge based on our previous findings^33^ and its involvement in APP processing and Aβ production^53^; however, other cytokines such as IL-1β and interferon-gamma have also been associated with Alzheimer’s disease pathology,^77^ and thus, it would be valuable to test them in future studies. Our results suggest a size-dependent aggregate clearance but do not exclude the possibility that all aggregates may be cleared initially, but some of the larger aggregates may then be preferentially taken-up by the neurons, accumulating inside. This possibility needs to be investigated in future work. Moreover, the 6E10 antibody used in this study has an epitope close to the N-terminal and binds to multiple Aβ fragments, as well as the βAPP C-terminal fragment and APP.^78^ While our aggregate-specific detection ensures the characterization of aggregated species with multiple epitopes, following-up on these results using Aβ40 and 42 specific antibodies would be helpful to gain further understanding on the aggregation and clearance of these peptides. The use of AT8 antibody in this study is also a potential limitation, since it restricts the tau aggregates that can be detected extracellularly. The AT8 antibody binds to mid-region of tau phosphorylated at residues Ser202 and Thr20578, present in the intracellular tau. However, the AT8 epitope is absent in the majority of the extracellular tau that consists of MTBR-containing aggregation-prone tau fragments. Our method therefore only detects AT8-positive tau aggregates formed inside the iPSC-derived neurons and released to the media, but not the aggregates formed directly by extracellular tau aggregation. Since the concentration of tau in the media is significantly lower than that in the neurons,^73^ tau aggregation directly in the media is expected to be low. Nevertheless, it would still be beneficial to use additional tau antibodies targeting the aggregates formed extracellularly in future studies to more comprehensively characterize the tau aggregates. Lastly, while it was not possible in this study due to sample availability, it will also be informative to study aSyn aggregates, since some discrepancies between our results here and our previous work^33^ might be due to changes in the number of aSyn aggregates with TNF-α treatment.

## Supplementary Material

fcae454_Supplementary_Data

## Data Availability

Data collected during these experiments will be made available upon reasonable request from the corresponding author. No novel code or software was generated to collect or analyse the data.
