# Supplementary material for: Clearance of beta-amyloid and tau aggregates is size dependent and altered by an inflammatory challenge
Source: Brain Commun. 2024 Dec 14;7(1):fcae454. doi: 10.1093/braincomms/fcae454 (PMC11694676; doi:10.1093/braincomms/fcae454)
Supplement: fcae454_Supplementary_Data [file fcae454_supplementary_data.pdf]

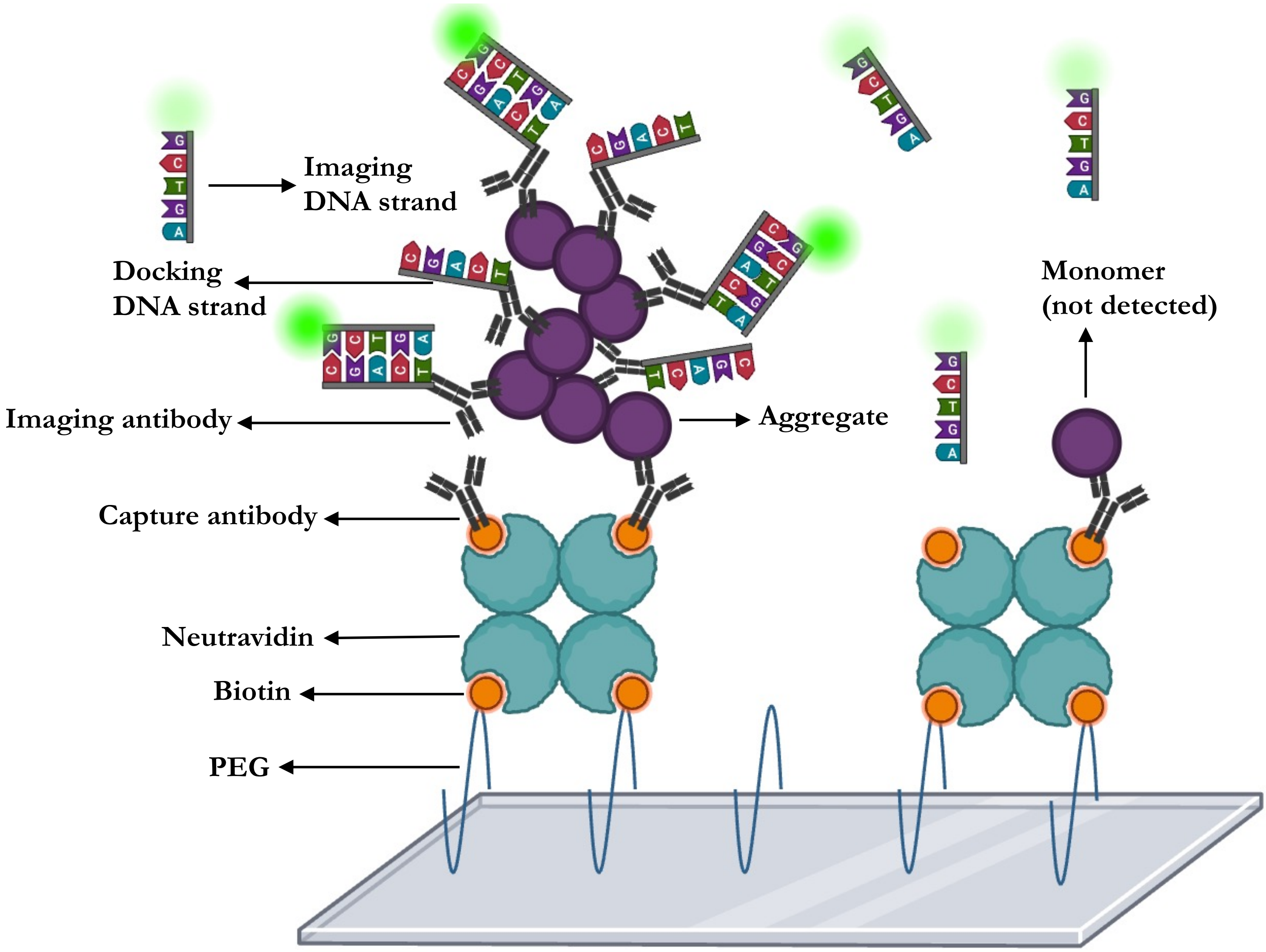

Supplemental Figure 1. Schematic representation of single-molecule pulldown (SiMPull) and DNA Point Accumulation in Nanoscale Topography (DNA-PAINT) imaging. The glass surface is pacified with PEG's attached to biotin. When neutravidin is added, it binds to biotin, ensuring the selective capturing of biotinylated antibodies. Targets of interest (Aβ and AT8+ tau) are captured by these antibodies. Same antibody is used for detection, enabling the selective imaging of aggregates (instead of monomers). The imaging antibody is conjugated to a single strand DNA and the complimentary strand is conjugated to the fluorophore, causing stochastic binding-unbinding events, which are used to localise and super-resolve the aggregates. A resolution of 20 nanometres is achieved. Generated with BioRender (Agreement number: IA27IW2SP4).

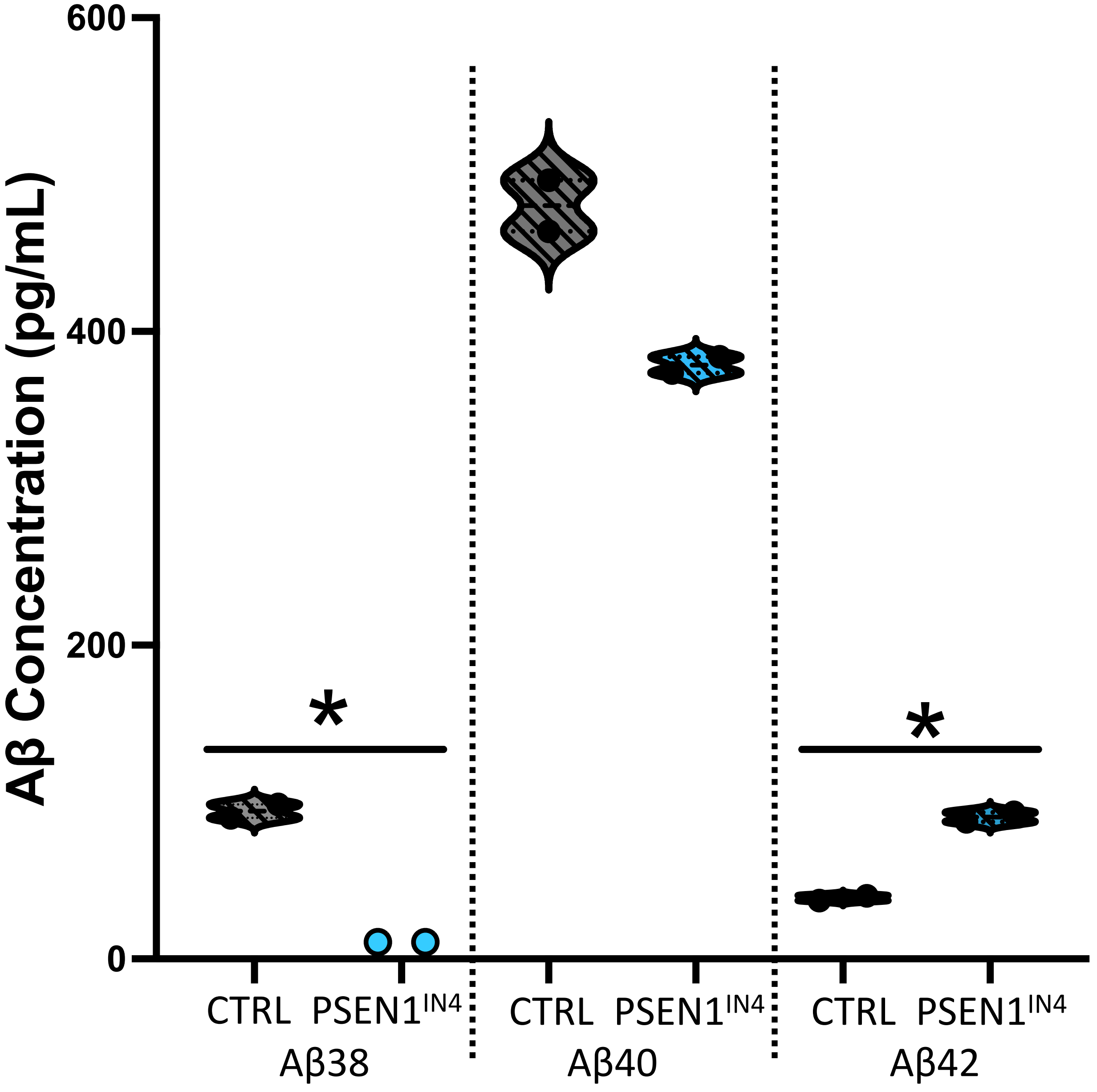

Supplemental Figure 2. Concentration of total Aβ38, 40, and 42 measured in conditioned media samples from the PSEN1^{IN4} and IC neurons prior to TNF-α treatment, measured by ELISA using two independent iPSC lines per genotype and two technical replicates, reported as picograms per millilitre (pg/mL). Three technical replicates from the ELISA were averaged for the independent iPSC lines. The Welch t-test showed a decrease in Aβ38 ($t_{2.01} = 13.75$, $p = 0.005$, $CI_{95} = 54.16$, 103.03), no difference in Aβ40 ($t_{2.14} = 0.82$, $p = 0.496$, $CI_{95} = -306.31$, 203.75), and an increase in Aβ42 levels ($t_{3.39} = 13.29$, $p < 0.001$, $CI_{95} = 39.31$, 62.07).

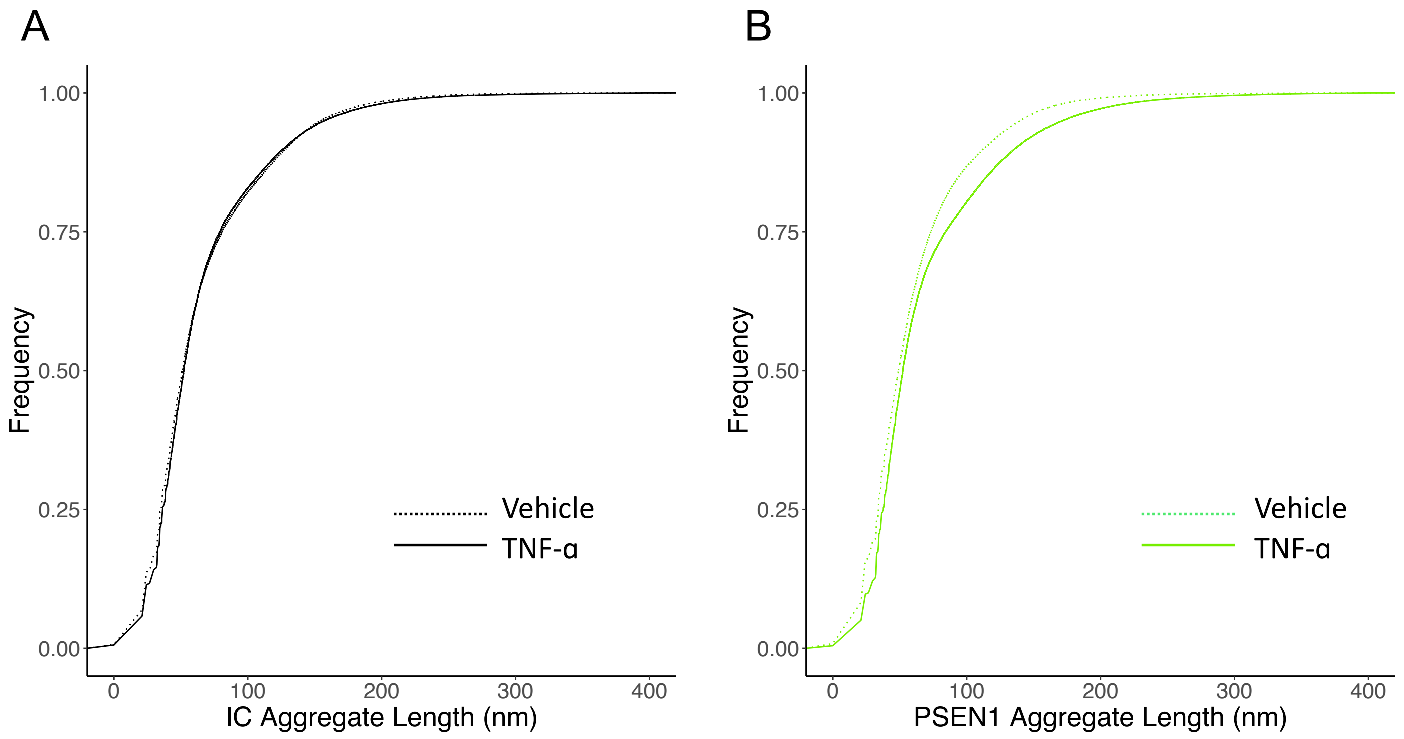

Supplemental Figure 3. Cumulative length distribution for IC (**A**) and PSEN1^{IN4} (**B**) neurons treated with vehicle or TNF-α shows a greater increase in treatment-dependent aggregate size in the PSEN1^{IN4} neurons. Sub-figure A is generated using data from 136,240 aggregates and Subfigure B is generated using data from 162,224 aggregates.

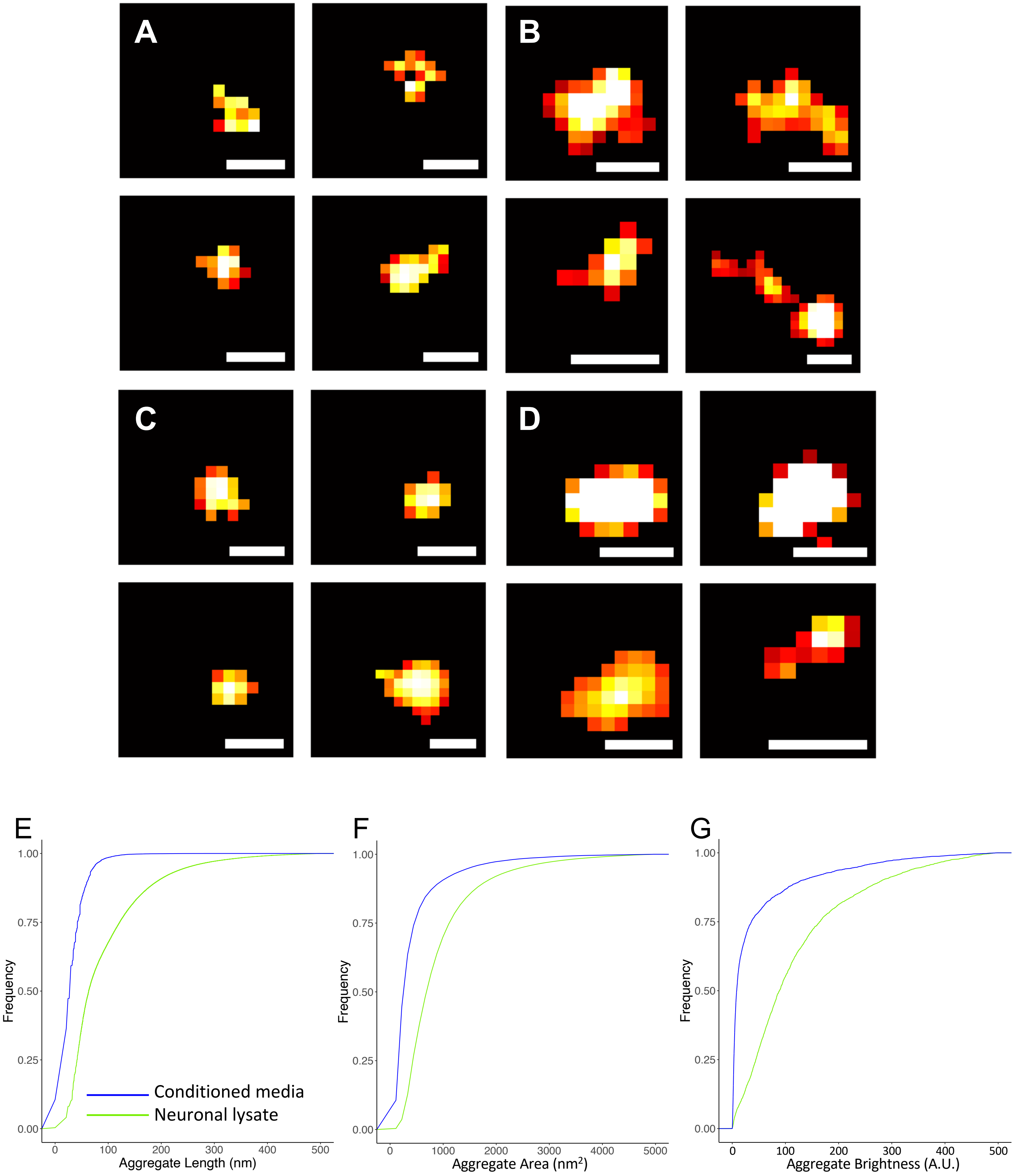

Supplemental Figure 4. Sample images of Aβ aggregates from IC (**A,B**) and PSEN1^{IN4} (**C,D**) hiPSC-derived cortical neurons conditioned media (**A,C**) and lysates (**B,D**) on DIV 60 (scale bars are 50 nm). Cumulative length (**E**) and area (**F**) distributions for hiPSC lysate and conditioned media samples show smaller Aβ aggregates are cleared from the hiPSC-derived cortical neurons while larger Aβ aggregates remain inside. Brightness analysis for AT8-positive tau shows a similar size-dependent clearance (**G**). Sub-figures E and F are generated using data from 519, 999 aggregates, and Subfigure G is generated using data from 7,505 aggregates.
